# Evaluation of primary breast cancers using dedicated breast PET and whole-body PET

**DOI:** 10.1038/s41598-020-78865-3

**Published:** 2020-12-14

**Authors:** Deep K. Hathi, Wen Li, Youngho Seo, Robert R. Flavell, John Kornak, Benjamin L. Franc, Bonnie N. Joe, Laura J. Esserman, Nola M. Hylton, Ella F. Jones

**Affiliations:** 1 Department of Radiology and Biomedical Imaging, University of California, San Francisco, CA USA; 2 Department of Epidemiology and Biostatistics, University of California, San Francisco, CA USA; 3 Department of Radiology, Stanford University, Stanford, CA USA; 4 Department of Surgery and Carol Franc Buck Breast Care Center, University of California, San Francisco, CA USA

**Keywords:** Biomarkers, Medical research, Cancer, Breast cancer, Cancer imaging

## Abstract

Metabolic imaging of the primary breast tumor with ^18^F-fluorodeoxyglucose ([^18^F]FDG) PET may assist in predicting treatment response in the neoadjuvant chemotherapy (NAC) setting. Dedicated breast PET (dbPET) is a high-resolution imaging modality with demonstrated ability in highlighting intratumoral heterogeneity and identifying small lesions in the breast volume. In this study, we characterized similarities and differences in the uptake of [^18^F]FDG in dbPET compared to whole-body PET (wbPET) in a cohort of ten patients with biopsy-confirmed, locally advanced breast cancer at the pre-treatment timepoint. Patients received bilateral dbPET and wbPET following administration of 186 MBq and 307 MBq [^18^F]FDG on separate days, respectively. [^18^F]FDG uptake measurements and 20 radiomic features based on morphology, tumor intensity, and texture were calculated and compared. There was a fivefold increase in SUL_peak_ for dbPET (median difference (95% CI): 4.0 mL^−1^ (1.8–6.4 mL^−1^), *p* = 0.006). Additionally, spatial heterogeneity features showed statistically significant differences between dbPET and wbPET. The higher [^18^F]FDG uptake in dbPET highlighted the dynamic range of this breast-specific imaging modality. Combining with the higher spatial resolution, dbPET may be able to detect treatment response in the primary tumor during NAC, and future studies with larger cohorts are warranted.

## Introduction

Breast cancer is a heterogeneous disease defined by the underlying genomic and proteomic expression profiles that affect treatment outcomes, recurrence risk, and response to therapy^1–4^. The identification of the four primary subtypes has enabled increasingly personalized treatment tailored to the molecular signature of breast cancers, resulting in improved prognoses and quality of life^5,6^. In particular, imaging of the primary tumor may provide critical predictive information for early response to targeted systemic therapies in patients receiving neoadjuvant chemotherapy (NAC)^7,8^.

Uptake of the glucose analog ^18^F-fluorodeoxyglucose ([^18^F]FDG) in tumor tissues provides an opportunity to assess the tumor’s viability and glycolytic potential. Whole-body positron emission tomography (wbPET) with [^18^F]FDG is the primary tool for disease staging, with demonstrated sensitivity in detecting early response to NAC^9–12^. However, the effectiveness of wbPET in the quantitative measurement of [^18^F]FDG uptake in primary breast tumors is hampered due to significant partial volume effects caused by limited spatial resolution, especially in small tumors^13,14^. Additionally, wbPET is primarily performed with the patient in a supine position, resulting in the collapse of the breast volume and blurring due to respiratory motion^15^. A complementary method for functional 3D imaging of the primary breast tumor is the use of dedicated breast PET (dbPET)^16^.

Studies with [^18^F]FDG-dbPET have qualitatively demonstrated its improved spatial resolution and uptake sensitivity within the primary tumor, at the expense of not being able to image metastatic lesions^17,18^. Compared to wbPET-CT, dbPET may also provide an opportunity for longitudinal imaging using a lower dose of the radiotracer (185 vs. 370 MBq) and lower radiation exposure without CT. The dbPET scanner in the current study consists of a single ring detector that translates axially over the length of the breast^19,20^. Because the patient is in a prone position, there is no breast compression, allowing for full breast volume imaging. We have also previously demonstrated this system's robustness for experimental imaging tracers, such as the radiotracer ^18^F-fluoroestradiol in a pilot study of estrogen receptor-positive breast cancer patients^21^. However, there is a need for validating the uptake, and tumor volume measurements generated from dbPET against the standard, clinically recognized wbPET. In this study, we assessed [^18^F]FDG uptake in pre-treatment breast cancer patients that received both dbPET and wbPET to characterize the similarities and differences in standard descriptive metrics, including standardized uptake values (SUVs), metabolic measures, and spatial heterogeneity statistics.

## Results

### Patient and tumor characteristics

This cohort consisted of ten patients with biopsy-confirmed, locally advanced, stage II-III invasive ductal carcinoma (Table 1). Patients in the study cohort had a median age of 46.5 with an interquartile range (IQR) of 39.3–57.8. 60% of the patients were pre-menopausal (≤ 50 years, N = 6), and 40% were post-menopausal (> 50 years, N = 4). Based on the hormone receptor (estrogen receptor (ER), progesterone receptor (PR)), and human epidermal growth factor receptor 2 (HER2) status, the primary breast tumors (N = 10) were mostly hormonal positive (ER+/PR+/HER2+ and ER+/PR+/HER2−, N = 6, 60%) and triple-negative (ER−/PR−/HER2−, N = 3, 30%). The median longest diameter measured by magnetic resonance imaging (MRI) was 3.4 cm (IQR 2.4–5.6 cm). The majority of patients (80%, N = 8) also had positive nodal involvement.

**Table 1:** Summary of patient and tumor characteristics.

Characteristics	Value
Number of patients (N)	10
**Tumor characteristics**	
Number of unique tumors (N)	10
Longest diameter by MRI (cm) [median (interquartile range)]	3.4 (2.3–5.6)
Stage II (N)	6
Stage III (N)	4
Age (years) [median (interquartile range)]	46.5 (39.3–57.8)
Body weight (kg) [median (interquartile range)]	66.6 (63.0–68.2)
**Menopause status (N)**	
Pre-menopause (< 50 years)	6
Post-menopause (> 50 years)	4
**Subtypes (N)**	
ER+/PR+/HER2+	2
ER+/PR+/HER2−	4
ER−/PR−/HER2+	1
ER−/PR−/HER2−	3
**Nodal status**	
Number of positive nodes (N)	15
Patients with positive involvement (%)	80%
Patients without positive involvement (%)	20%

*ER* estrogen receptor, *PR* progesterone receptor, *HER2* human epidermal growth factor-2, *MRI* magnetic resonance imaging.

### *Comparison of [*^*18*^*F]FDG-dbPET and [*^*18*^*F]FDG-wbPET*

**Figure 1:**
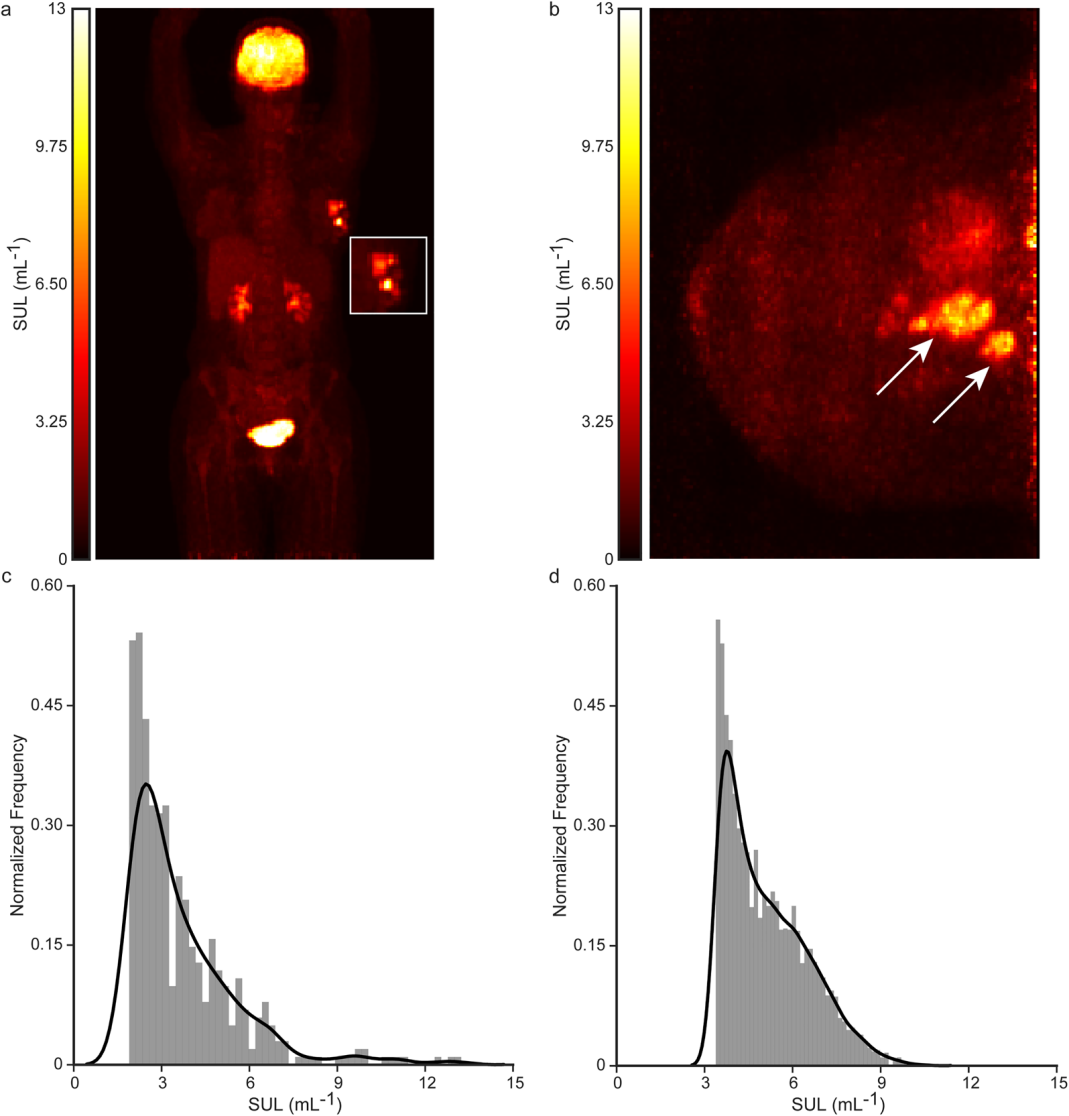
An example of a 39-year-old breast cancer patient with an agglomerate of ER+/PR+/HER2+ tumors imaged by [^18^F]FDG-wbPET and -dbPET at the pre-treatment timepoint. (**a**) A maximum intensity projection of [^18^F]FDG-wbPET with primary lesions in the left ipsilateral breast (inset). (**b**) A maximum intensity projection of [^18^F]FDG-dbPET with corresponding highlighted tumors in the left ipsilateral breast (white arrows). (**c**) Histogram of SULs in the tumor VOI for wbPET. (**d**) Histogram of SULs in the tumor VOI for dbPET. Number of bins in (**c**,**d**) were restricted to 50.

Representative [^18^F]FDG-wbPET and -dbPET images of a 39-year-old breast cancer patient with an agglomerate of ER+/PR+/HER2+ tumors are shown in Fig. 1. The primary tumor was highlighted in the left breast, with comparable maximum standardized uptake value normalized to lean body mass (SUL_max_) for both methods (wbPET 13.25 mL^−1^ vs dbPET 10.57 mL^−1^). The primary difference observed between the dbPET and wbPET was the heterogeneity of the tumor in the ipsilateral breast. Due to the lower post-reconstruction voxel resolution (4 $\times$ 4 $\times$ 4 mm) and the smaller breast tumor tissue size relative to the total field of view (FOV), the primary tumor in the wbPET comprises a relatively small fraction of the total voxel volume (Fig. 1a, a inset). By contrast, dbPET had a higher in-tumor resolution (1 $\times$ 1 $\times$ 1 mm post-reconstruction) and highlighted the spatial heterogeneity (Fig. 1b). Signal intensity histograms of the tumor volume of interest (VOI) with fixed bin number (50 bins) were generated for wbPET (Fig. 1c) and dbPET (Fig. 1d). The observed qualitative differences in spatial and signal intensity heterogeneity in dbPET may be largely driven by the higher voxel resolution and tumor tissue fraction.

**Figure 2:**
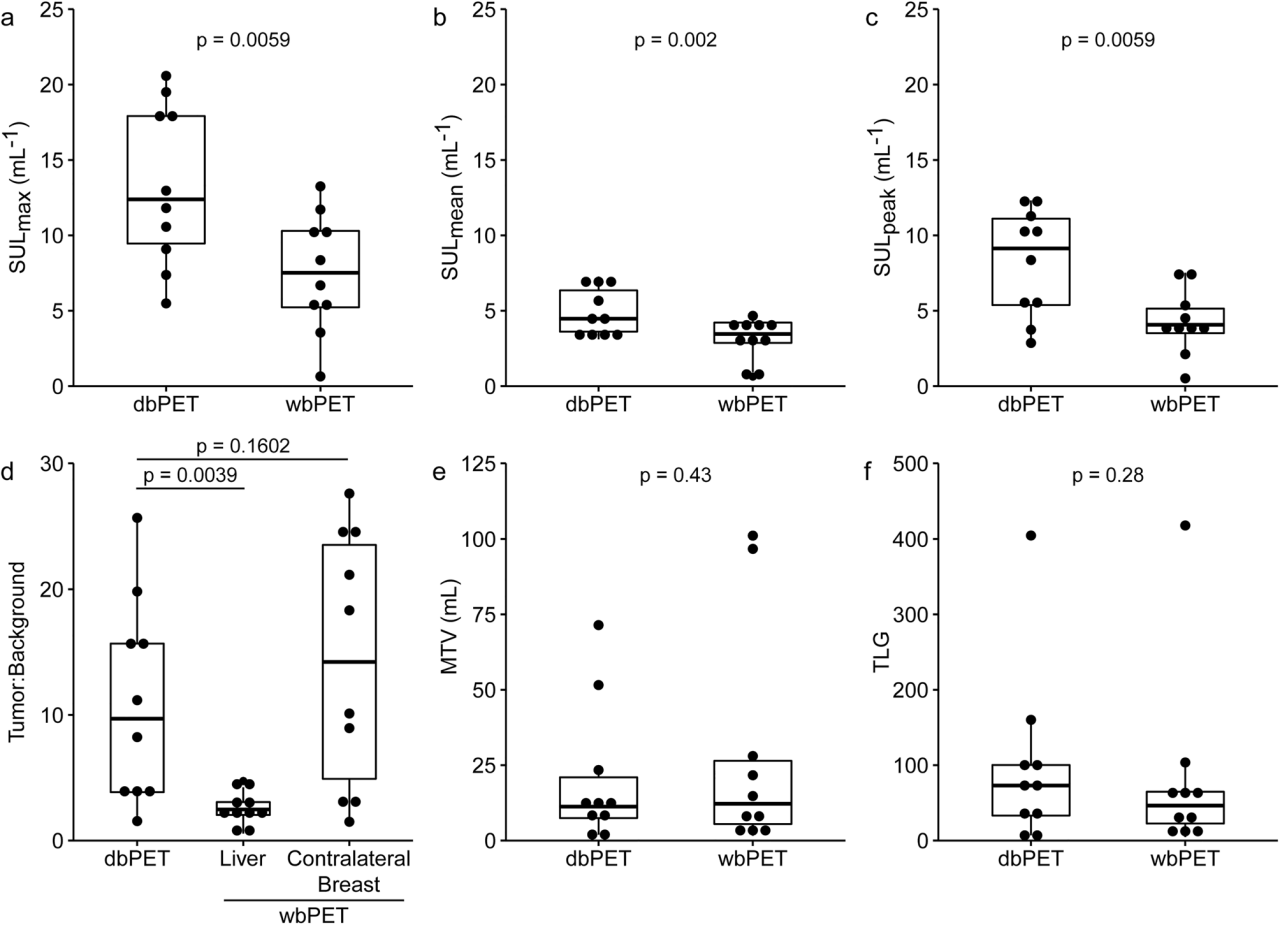
Comparison of [^18^F]FDG uptake in lesions with dbPET and wbPET. (**a**) SUL_max_; (**b**) SUL_mean_; (**c**) SUL_peak_; (**d**) Ratio of SUL_peak_ in tumor to SUL_mean_ in the background. For wbPET, the background VOIs were defined separately on the liver and the contralateral breast. Background VOI was defined in the contralateral breast for dbPET. (**e**) Metabolic tumor volume (MTV); and (**f**) Total lesion glycolysis (TLG).

Uptake and metabolically active tumor volume statistics were calculated and compared for both modalities (Fig. 2). SUL_max_ (median difference (95% CI): 5.88 mL^−1^ (2.88, 8.87 mL^−1^), *p* = 0.006), SUL_mean_ (1.76 mL^−1^ (0.89, 2.58 mL^−1^), *p* = 0.002), and SUL_peak_ (4.01 mL^−1^ (1.80, 6.40 mL^−1^), *p* = 0.006) were all statistical significantly higher for dbPET relative to wbPET (Fig. 2a–c). The tumor-background ratios with contralateral breast normalization were similar [− 2.28 (− 9.14, 1.56), *p* = 0.16] for both modalities, although normalization of the wbPET tumor uptake by the liver VOI resulted in a statistically significant higher tumor-background ratio in dbPET relative to wbPET [7.87 (2.17, 13.71), *p* = 0.004]. The metabolic tumor volume (MTV) was reduced in dbPET [− 4.62 mL (− 23.91, 1.72 mL), *p* = 0.43], while the total lesion glycolysis (TLG) was higher in dbPET [17.63 (− 8.41, 49.15), *p* = 0.27] (Fig. 2e,f). The broad distribution of uptake and tumor volume statistics may be driven by inter-patient heterogeneity.

Since the partial volume effect is driven by the lesion size and spatial resolution of reconstructed images, the cohort was also examined based on the MRI longest diameter (*d*_MRI_) with a cutoff of 2.5 cm [median (IQR) 2.23 (2.2–2.33) cm, N = 4; 5.45 (3.9–6.48) cm, N = 6] (Table 2). There was a 2.71-fold and 1.58-fold increase in SUL_peak_ in dbPET compared to wbPET in the *d*_MRI_ ≤ 2.5 cm and *d*_MRI_ > 2.5 cm groups, respectively. The smaller difference in uptake for the lesions with *d*_MRI_ > 2.5 cm was expected as the impact on partial volume effect due to the spatial resolution difference between dbPET and wbPET is smaller for larger lesions^14,22^. The differences between dbPET and wbPET in the uptake (SUL) and volume-derived measurements (MTV, TLG) generally increased in the smaller lesions (*d*_MRI_ ≤ 2.5 cm) than larger tumors (*d*_MRI_ > 2.5 cm).

**Table 2:** Summary of dbPET and wbPET uptake metrics for MRI longest diameter (*d*_MRI_) ≤ 2.5 cm and *d*_MRI_ > 2.5 cm.

	dbPET [Median (IQR)]		wbPET [Median (IQR)]	
	*d*_MRI_ ≤ 2.5 cm	*d*_MRI_ > 2.5 cm	*d*_MRI_ ≤ 2.5 cm	*d*_MRI_ > 2.5 cm
SUL_max_	12.39 (10.23–14.6)	14.22 (9.46–17.92)	7.52 (5.17–8.78)	8.04 (5.23–11.38)
SUL_mean_	5.78 (4.37–7.01)	4.00 (3.45–5.32)	3.56 (2.65–4.08)	3.35 (2.87–4.18)
SUL_peak_	11.05 (8.15–12.22)	7.11 (5.38–10.05)	4.07 (3.07–4.29)	4.50 (3.51–6.81)
Tumor-Background (Liver)	N/A	N/A	2.39 (1.87–2.64)	2.71 (2.07–3.92)
Tumor-Background (Contralateral breast)	4.01 (3.65–7.17)	13.41 (8.97–18.78)	16.64 (7.09–24.44)	14.22 (5.20–20.45)
MTV	9.00 (5.39–12.02)	17.25 (10.39–44.53)	5.8 (4.26–7.26)	24.85 (16.49–79.56)
TLG	54.84 (24.51–84.43)	83.57 (47.66–145.2)	25.06 (16.19–30.34)	64.53 (62.24–95.4)

Finally, 20 tumor morphology, tumor signal intensity, and textural features were generated from both modalities (Table 3). Spatial heterogeneity features, specifically the gray-level co-occurrence matrix (GLCM) correlation [median difference (95% CI) − 0.42 (− 0.60, − 0.25), *p* = 0.002], normalized inverse difference [− 0.05 (− 0.08, − 0.02), *p* = 0.002], and joint entropy [− 0.71 (− 2.30, − 0.15), *p* = 0.01], showed statistically significant difference between dbPET and wbPET. There was also a statistically significant decrease in mesh volume [− 11.00 mL (− 22.59, − 2.13 mL), *p* = 0.006] in dbPET relative to wbPET.

**Table 3:** Summary of radiomics features for dbPET and wbPET.

	Features	dbPET Median (IQR)	wbPET Median (IQR)	Median difference (95% CI)	P-Value
Morphology	Mesh volume (cm^3^)	6.08 (2.94–13.65)	19.19 (7.36–26.87)	− 11.00 (− 22.59, − 2.13)	**0.006**
	Surface area (cm^2^)	26.12 (15.46–68.29)	51.59 (25.28–92.57)	− 17.76 (− 150.57, 2.66)	0.77
	Sphericity	0.65 (0.54–0.69)	0.70 (0.58–0.74)	− 0.03 (− 3.06, 0.90)	0.43
	Maximum 3D Diameter (cm)	4.24 (2.63–4.80)	43.72 (34.37–72.71)	− 1.13 (− 43.07, 0.22)	0.28
Tumor Intensity	Entropy	7.96 (7.49–9.12)	8.89 (8.02–10.33)	− 1.12 (− 2.53, 0.3)	0.28
	Uniformity	5.1e−3 (2.1e−3–6.5e−3)	2.4e−3 (9.5e−4–4.6e−3)	6.3e−4 (− 0.003, 0.005)	0.70
	Skewness	0.90 (0.83–1.07)	0.59 (0.43–0.76)	0.32 (− 0.11, 0.65)	0.13
	Kurtosis	3.31 (2.74–3.62)	2.69 (2.57–3.31)	0.53 (− 0.63, 2.93)	0.19
	Standard Deviation	1.42 (0.99–2.09)	1.7 (1.22–2.82)	− 0.60 (− 3.06, 0.90)	0.43
GLCM	Contrast	2.6e4 (1.4e4–7.7e4)	1.9e4 (7.2e3–2e5)	6.8e3 (− 3.6e5, 1.1e5)	0.92
	Correlation	0.43 (0.27–0.54)	0.78 (0.76–0.84)	− 0.42 (− 0.60, − 0.25)	**0.002**
	Inverse Difference	0.03 (0.02–0.04)	0.03 (0.01–0.05)	− 0.01 (− 0.11, 0.01)	0.56
	Normalized Inverse Difference	0.87 (0.84–0.90)	0.92 (0.90–0.93)	− 0.05 (− 0.08, − 0.02)	**0.002**
	Joint Energy	9.01e−4 (4.5e−4–0.002)	6.5e−4 (2.4e−4–9.7e−4)	5.14e−4 (− 1.86e−5, 8.37e−3)	0.084
	Joint Entropy	10.17 (8.93–11.13)	10.97 (10.03–12.06)	− 0.71 (− 2.30, − 0.15)	**0.01**
NGTDM	Busyness	0.008 (0.003–0.019)	8.2e−4 (2.8e−4–1.4e−3)	0.004 (− 0.04, 0.01)	0.0840
	Coarseness	0.003 (0.002–0.007)	0.006 (0.003–0.009)	− 0.002 (− 0.005, 0.007)	0.2324
	Complexity	4.6e6 (2e6–3.7e7)	8.7e6 (1.7e6–2.8e8)	− 4.69e6 (− 1.97e9, 5.37e7)	0.7695
	Contrast	31.52 (10.06–116.78)	10.8 (8.05–49.11)	42.24 (− 26.82, 242.48)	0.2754
	Strength	490.9 (248.9–1.6e3)	2.4e3 (1.4e3–6.6e3)	− 0.001 (− 1.4e4, 176.6)	0.1602

Statistical significance, median difference, and 95% confidence intervals were assessed using the Wilcoxon signed-rank test.

*GLCM* gray-level co-occurrence matrix, *NGTDM* neighborhood gray-tone difference matrix.

## Discussion

The motivation for this study was to compare the performance of primary tumor characterization between [^18^F]FDG-dbPET and [^18^F]FDG-wbPET in a pilot study of breast cancer patients. WbPET is primarily used for staging and can detect metastases and nodal involvement, which are necessary to devise treatment strategies. However, due to the collapsed breast volumes and low relative tumor tissue fraction, wbPET may be limited in precise and quantitative characterization of the primary tumor for studying therapeutic response. Here, we examine the similarities and differences in the distribution of [^18^F]FDG uptake in dbPET and wbPET at the pre-treatment time point.

Steady-state uptake metrics were used to characterize [^18^F]FDG-wbPET and -dbPET. As suggested by the PET Response Criteria in Solid Tumors (PERCIST), the lean body mass was used to generate SULs to account for the dual effects of body weight and height on uptake^23^. In general, overall [^18^F]FDG uptake values (SUL_max_, SUL_mean_, and SUL_peak_) in the primary tumor were higher in dbPET compared to wbPET. SUL_peak_ is considered more robust than SUL_max_ and SUL_mean_ due to its insensitivity to voxel-wise variations. The higher dbPET SUL_peak_ reflects the sensitivity and dynamic range required for detecting changes in tumor uptake following treatment, although the relative increase in the dynamic range and sensitivity is likely paired with increased background noise. To minimize the effect of background noise, a threshold for dbPET with SUV ≥ 3.0 was applied to define tumor boundaries. The calculation of SUL_peak_ involves mean filtering with a 1.2 cm spherical kernel, which may further reduce the effect of the increased background signal on the presented SUL_peak_. Although the results did not reach statistical significance, MTV and TLG were estimated to be higher in wbPET compared to dbPET. These trends suggest a role for the partial volume effect, due to lower spatial resolution and tumor tissue fraction in wbPET^14^. Post hoc analysis of SULs and tumor volume supports this analysis, with the observed increases in dbPET-measured SUL_peak_, MTV, and TLG in lesions with *d*_MRI_ ≤ 2.5 cm.

To account for variability introduced by the surrounding tissue, the tumor signal is often normalized by the background VOI. In [^18^F]FDG-wbPET, the background VOI is defined in the liver in the absence of liver metastases, due to its perfused and homogenous uptake of [^18^F]FDG. However, this normalization method was not available for dbPET and the contralateral breast was used. This method resulted in a fourfold increase of tumor-background ratio in dbPET compared to wbPET using liver as the background. While the contralateral tissue is a logical background tissue for dbPET, careful consideration should be taken if there is bilateral disease. We observed inter-patient heterogeneity in the contralateral breast background signal in both modalities, which resulted in minimal difference in tumor-background ratio following normalization by the contralateral breast in wbPET. Similar variation in background parenchymal FDG uptake have been observed in other studies^24,25^. While limiting the PET scans at the early follicular phase of the menstrual cycle may minimize the background parenchymal signal^26^, the patient’s treatment planning should be taken into consideration for scheduling without imposing additional burden. Finally, radiomic features pertaining to heterogeneity and tumor morphology were calculated within the tumor VOI for dbPET and wbPET. The improved spatial resolution within the primary breast tumor and increased tumor tissue fraction in dbPET relative to wbPET have been previously reported, and qualitatively represented in an increase of metabolic heterogeneity in the tumor in a cohort of 35 patients^27^. While textural features of the primary tumor from dbPET images have been analyzed based on breast cancer subtypes^28^, the similarities and differences of these features have not been assessed between dbPET and wbPET. The chosen features have been identified in the literature to be most reflective of changes in spatial and intensity heterogeneity^29^ and robust to imaging conditions and reconstruction parameters^30^. To prevent resolution biases from affecting feature calculations, dbPET and wbPET images were down-sampled and up-sampled, respectively, to an isotropic voxel size (2 $\times$ 2 $\times$ 2 mm). Morphological features were concordant with standard uptake volume metrics (MTV and TLG), especially mesh volume, which was found to be significantly different between the two modalities. Statistically significant difference was also observed in features corresponding to spatial heterogeneity, specifically GLCM normalized inverse difference, joint entropy, and correlation. The differences in textural heterogeneity features correlate to the observed, qualitative differences between dbPET and wbPET and may be primarily driven by the increased sensitivity in dbPET.

The [^18^F]FDG-dbPET results in this study are concordant with literature of positron emission mammography (PEM, NaviScan, CA) and previous cohorts with the MAMMI and Elmammo (Shimadzu, Japan) dbPET systems. In a cohort of 388 patients with primary breast lesions, PEM showed comparable accuracy to MRI with improved sensitivity compared to wbPET for quantifying primary lesions^31,32^. Similar to dbPET, PEM had a smaller FOV focusing on the primary lesion at the expense of metastatic lesions, although breast compression and hardware design resulted in limited resolution with maximum intensity projection of 2D images^16^. The sensitivity, spatial resolution, and dose for dbPET in our study recapitulated observations in a cohort of 234 breast cancer patients who received [^18^F]FDG-dbPET and wbPET/CT for staging^17^. In that study, sub-centimeter lesions in the ipsilateral breast were identified by dbPET that were not resolved in the wbPET images. Similarly, using the Elmammo system, Sasada et al. compared bilateral [^18^F]FDG-dbPET to [^18^F]FDG-wbPET in a 47-patient cohort following NAC and observed that the tumor-background ratio was more sensitive than SUV_max_ to treatment response^33^. Nishimatsu et al*.* also observed higher tumor-background ratios in dbPET relative to wbPET in a cohort of 150 patients, although there was no observable difference in sensitivity to lesion detection^34^. In our study, the SUL_peak_ was higher in dbPET, suggesting a larger dynamic range and potentially, higher sensitivity to treatment-induced changes in [^18^F]FDG uptake. Furthermore, analysis of radiomic features identified statistically significant differences in GLCM-derived textural heterogeneity markers between dbPET and wbPET.

The limitations of our study are largely driven by hardware design and cohort construction issues. Lesions adjacent to the chest wall experienced limited resolution and incidental scatter from the heart, resulting in inaccurate quantification of uptake. This issue may be mitigated in future studies by using an enlarged aperture on the scan bed, a thinner chest resting area, and a flexible silicone sleeve to gain more breast tissues near the chest wall to be scanned within the detector field of view. An improved reconstruction algorithm as described by O’Connor et al*.*^18^ will be installed to reduce the presence of crosstalk in the breast from FDG signals in the myocardium.

In addition, this pilot study had a limited sample size that prohibited additional analyses based on breast cancer subtypes. While our objective was to compare the [^18^F]FDG signal distribution in dbPET and wbPET, results from this small cohort cannot be generalized. Study inclusion criteria placed a lower bound on tumor sizes at 2 cm, which limited the study of smaller lesions with high potential for partial volume effect. Since the wbPET was part of a routine clinical procedure, wbPET data included in this report were acquired using different systems with various reconstruction algorithms, depending on clinical availability. SUVs generated across vendors at multiple sites are known to possess coefficients of variation of up to 5%, although this error may be reduced through frequent calibrations^35^. The different wbPET scanners and reconstruction algorithms may also affect radiomics feature stability. To prevent undue adverse effect on reproducibility for wbPET features, we selected more robust and repeatable GLCM features^36,37^ in this study. Additionally, tumor-masked images were rescaled to an isotropic resolution and quantized to discrete gray-levels with a fixed bin width^38^ to prevent intensity-driven heterogeneity from affecting the radiomics results. Finally, the patient cohort was mostly locally advanced stage II/III patients from a single institution, which may not be representative of the general breast cancer patient population.

In this study, we assessed similarities and differences in [^18^F]FDG uptake between wbPET and dbPET. SUL_peak_ and SUL_max_ are well recognized as predictors of treatment response and showed consistently higher values measured by dbPET compared to wbPET. While dbPET is not designed to detect metastatic disease for staging, it serves as an adjunct to wbPET and breast MRI for breast tumor characterization^20^. Compared to wbPET, the higher dbPET readout also reflects the higher sensitivity and broader dynamic range for detecting treatment-induced changes in the primary non-metastatic tumor, providing powerful molecular insights to guide treatment selection and to better assess early molecular changes in response to treatment.

The increased spatial resolution and reduced [^18^F]FDG dose used in dbPET makes it an attractive modality for treatment monitoring and supports further analyses using higher-order radiomic features to quantify changes in tumor burden and intratumoral heterogeneity for treatment stratification and prediction of survival outcomes. Future studies with this technology would utilize larger cohorts and receptor-specific radiotracers to improve stratification.

## Methods

### Ethics statement

Ten patients with biopsy-confirmed breast cancer were recruited to participate in an imaging study with [^18^F]FDG-dbPET (MAMMI, General Equipment and Medical Imaging SA (OncoVision), Valencia, Spain). This study was in compliance with the HIPAA-compliant study protocol that was reviewed by the UCSF Institutional Review Board and approved by the Committee of Human Research under the institution Human Research Program. All procedures performed were in compliance with relevant guidelines and regulations. All patients were required to provide written informed consent to participate.

### Study population

Patient eligibility was established on the basis of histopathological evaluation of biopsy samples. Breast MRI was performed as a standard of care to measure the extent of the tumor burden and size, while [^18^F]FDG-wbPET was used for staging.

### *[*^*18*^*F]FDG-dbPET acquisition*

DbPET was performed with [^18^F]FDG (range 121.0–199.8 MBq, median 185.7 MBq). Patients fasted 6 h prior to imaging and blood glucose was measured before intravenous [^18^F]FDG administration. Following 45 min of incubation, the subjects were scanned in a prone position, with a single breast positioned through the aperture into the detector ring. The detector ring translated axially from inferior to superior for approximately 15 min, with the frame duration determined by the total length of the breast. DbPET images were reconstructed in 3D using manufacturer-provided maximum-likelihood expectation maximization with a 1 mm kernel and 16 iterations. The post-reconstruction resolution was standardized to 1 $\times$ 1 $\times$ 1 mm. Both breasts were scanned consecutively in the same imaging session.

### *[*^*18*^*F]FDG-wbPET acquisition*

WbPET with computed tomography (wbPET/CT) was performed as part of disease staging and routine clinical procedure. As with dbPET, patients fasted for approximately 6 h prior to the scan, and blood glucose levels were measured before intravenous [^18^F]FDG administration (range 229.4–392.6 MBq, median 301.6 MBq). After approximately 50–60 min of incubation, patients were scanned in the supine position through the PET/CT gantry, translating anterior to posterior for approximately 30 min. Depending on the clinical availability, wbPET data included in this report were acquired using three PET/CT scanners: GE Discovery VCT PET/CT (GE Healthcare, WI), Philips Gemini TF PET/CT, and Philips Vereos PET/CT (Philips Healthcare, MA). All wbPET images were acquired in 3D and reconstructed using manufacturer-provided iterative reconstruction algorithms that included CT-based attenuation and scatter corrections. The standard whole-body image reconstruction algorithms were: 3D ordered subsets expectation maximization (OS-EM) with 28 subsets and 2 iterations for GE Discovery VCT PET/CT; Blob-based iterative time-of-flight reconstruction algorithm (BLOB-OS-TF) with 3 iterations and 33 subsets for Philips Gemini TF PET/CT; Time-of-flight OS-EM with 3 iterations and 15 subsets for Philips Vereos PET/CT. The most common post-reconstruction voxel resolution across systems was 4 $\times$ 4 $\times$ 4 mm (other voxel resolutions in the study: 5.5 $\times$ 5.5 $\times$ 3.3 mm, 3.6 $\times$ 3.6 $\times$ 3.3 mm).

### PET data analysis

To compare wbPET and dbPET [^18^F]FDG uptake, reconstructed images were first converted to decay-corrected standardized uptake values normalized by body weight (SUV) and lean body mass (SUL). Semi-automated segmentation of the tumor volume of interest (VOI) was performed over the entire volume of the high uptake lesion (OsiriX, Pixmeo, Switzerland) with a threshold of SUV ≥ 2.5 and 3.0 for wbPET and dbPET, respectively. Background SUVs were measured by placing a 1.2 cm cylindrical mask at the centroid of the contralateral breast for dbPET and a 1.2 cm spherical mask in the liver and contralateral breast for wbPET. Following segmentation, the single voxel maximum, average, and peak uptake (SUL_max_, SUL_mean_, SUL_peak_) were computed as per the standard PET Response Criteria in Solid Tumors (PERCIST), while the metabolic tumor volume (MTV) was calculated as the summing of voxel volumes with SUV ≥ 40% SUV_mean_^39^. The total lesion glycolysis (TLG) was computed as the product of the MTV and SUV_mean_.

Radiomic features were computed within the tumor VOIs using Python 3.7. Images were first re-segmented in 3D Slicer 4.11^40^ and resampled to an isotropic resolution (2 $\times$ 2 $\times$ 2 mm^3^) using linear interpolation and quantized to discrete gray-levels using a fixed bin width^38,41^. Radiomics calculation was performed on these discretized images.

A total of 20 features describing tumor morphology and heterogeneity were calculated using the PyRadiomics package according to the Image Biomarker Standardization Initiative recommendations^42–44^. In particular, heterogeneity features were evaluated from intensity distribution and texture analysis, specifically the grey-level co-occurrence matrix (GLCM) and neighborhood grey-tone difference matrix (NGTDM)^45,46^. Prior to the calculation of textural features, SUV images were harmonized in accordance to literature methods^41^. The data were rescaled to discretized grey levels with a bin width of 0.5. GLCM and NGTDM features were calculated on the 3D volume masked by the tumor VOI with nearest neighbor distances (*d* = 1) and averaging of each angle-specific matrix. The GLCM and NGTDM classes describe neighboring pixel and regional textures, respectively.

All statistical analyses were performed using R v. 3.6.2 (R Foundation for Statistical Computing, Vienna, Austria). Statistical significance, median difference, and 95% confidence intervals (CI) were assessed using the non-parametric Wilcoxon signed-rank test. *p*-values less than 0.05 were considered significant.

### Consent to participate

All patients provided written informed consent to participate.

### Consent for publication

All patients provided written informed consent for publication.

## Data Availability

Data will be available upon request.
